# Comprehensive prediction of lncRNA–RNA interactions in human transcriptome

**DOI:** 10.1186/s12864-015-2307-5

**Published:** 2016-01-11

**Authors:** Goro Terai, Junichi Iwakiri, Tomoshi Kameda, Michiaki Hamada, Kiyoshi Asai

**Affiliations:** INTEC Inc, 1–3-3 Shinsuna Koto-ku, Tokyo, 136-8637 Japan; Graduate School of Frontier Sciences, University of Tokyo, 5-1-5 Kashiwanoha, Kashiwa, Chiba, 277–8562 Japan; Biotechnology Research Institute for Drug Discovery, National Institute of Advanced Industrial Science and Technology (AIST), 2-41-6, Aomi, Koto-ku, Tokyo, 135-0064 Japan; Faculty of Science and Engineering, Waseda University, 55N-06-10, 3-4-1, Okubo Shinjuku-ku, Tokyo, 169-8555 Japan

**Keywords:** RNA–RNA interactions, Interaction energy, TINCR, 1/2-sbsRNA

## Abstract

**Motivation:**

Recent studies have revealed that large numbers of non-coding RNAs are transcribed in humans, but only a few of them have been identified with their functions. Identification of the interaction target RNAs of the non-coding RNAs is an important step in predicting their functions. The current experimental methods to identify RNA–RNA interactions, however, are not fast enough to apply to a whole human transcriptome. Therefore, computational predictions of RNA–RNA interactions are desirable, but this is a challenging task due to the huge computational costs involved.

**Results:**

Here, we report comprehensive predictions of the interaction targets of lncRNAs in a whole human transcriptome for the first time. To achieve this, we developed an integrated pipeline for predicting RNA–RNA interactions on the K computer, which is one of the fastest super-computers in the world. Comparisons with experimentally-validated lncRNA–RNA interactions support the quality of the predictions. Additionally, we have developed a database that catalogs the predicted lncRNA–RNA interactions to provide fundamental information about the targets of lncRNAs.

**Electronic supplementary material:**

The online version of this article (doi:10.1186/s12864-015-2307-5) contains supplementary material, which is available to authorized users.

## Introduction

Non-coding RNAs (ncRNAs), which are not translated into proteins but play essential roles in various biological processes, have been receiving increased attention [1, 2]. Among them, long non-coding RNAs (lncRNAs) have turned out to be involved in development, differentiation, epigenetic regulation and the immune system [1–3], as well as to be related to disease [4]. There are more than 20,000 lncRNAs listed in Gencode [5], but the functions of only a few lncRNAs, such as those of Xist [6] and NEAT1 [7], have been experimentally verified.

Knowing which RNAs and/or proteins are the interaction targets is essential for determining the functions of lncRNAs. The ncRNAs whose functional mechanisms have been identified are known to interact with other RNAs and/or proteins. Therefore, identifying the target RNAs or proteins with which an lncRNA interacts is the first step in characterizing the function of an lncRNA.

Several lncRNA–RNA interactions have been verified by experiment. Gong and Maquat [8] investigated the lncRNA 1/2-sbsRNA, which interacts with the 3 ^′^UTR of two mRNAs, leading to Staufen 1 (STAU1)-mediated messenger RNA decay (SMD); [9] determined the interactome of the 3.7 kilo-base lncRNA, a terminal differentiation-induced ncRNA (TINCR), by using an experimental method called RIA-seq (RNA interactome analysis with new generation sequencers). Their results suggest that TINCR interacts with many mRNAs through a sequence motif. Abdelmohsen et al. [10] suggested that the lncRNA 7SL interacts with TP53 mRNA, which encodes the tumor suppressor p53. In all of the above cases, the interaction partners of the lncRNAs were mRNAs. However, it is natural to investigate the possibility of lncRNA-lncRNA interactions in light of external base-pairs. In this study, we therefore focus on both lncRNA–mRNA and lncRNA–lncRNA interactions.

The experimental methods proposed to investigate RNA–RNA interactions (e.g., [9, 11]) require a specific target RNA. A similar situation occurs in CLIP-seq for RNA-protein interactions, where the target protein should be specified [12]. Hence, it is quite laborious to comprehensively determine the interactome for a large scale transcriptome across all possible pairs of RNAs under a variety of conditions (e.g., tissue, cell-type and time). These limitations emphasize the need for computational prediction of RNA–RNA interactions.

For *in-silico* predictions of non-coding RNAs, a widely accepted software tool, RNAZ, has been applied to the human genome and had an impact on non-coding RNA research [13]. However, *in-silico* predictions of RNA–RNA interactions are limited to small datasets, such as bacterial *small* RNAs (sRNAs) [14–17] because the high computational cost of predicting RNA–RNA interactions prevents us from making comprehensive predictions due to the huge combinatorial number of candidate RNA pairs in the whole human transcriptome.

For predicting interactions between two RNA molecules, an understanding of the base-pair interactions between the two RNAs is essential [15]. RNA–RNA interactions, however, are not simple processes consisting of forming base-pairs between the two RNA molecules, even if only secondary structures are considered. The two RNA molecules form a *joint* secondary structure, which involves both intra-molecular and inter-molecular base-pairs. Computational prediction of the joint secondary structures of two RNAs of length *L* has time complexity *O*(*L*^4^) to *O*(*L*^6^), depending on the complexity of the structures considered. It becomes infeasible to perform this calculation for all *O*(*N*^2^) pairs of RNAs when *N* (the number of RNA sequences) becomes large.

In this study, we report comprehensive predictions of lncRNA–RNA interactions in the human transcriptome (including lncRNAs and mRNAs) for the first time. To achieve this, we have developed a fast pipeline for predicting RNA–RNA interactions for a large number of RNA sequences, and have implemented the pipeline on the K computer (http://www.aics.riken.jp/en/k-computer/about/), which is one of the fastest super-computers in the world.

In order to evaluate the proposed method, experimentally validated human lncRNA–RNA interactions were compared with the predictions of our pipeline. To avoid overfitting the pipeline to human transcriptome, the three adjustable parameters in our pipeline were determined by using the *E. coli* dataset as a training set. The results support the overall better performance of the pipeline compared with existing approaches.

As a further contribution for researchers studying ncRNAs, we have developed a database that contains all predicted lncRNA–RNA interactions; this is available for public use.

## Materials and methods

For comprehensive prediction of RNA–RNA interactions, it is necessary to reduce the heavy computational costs, both for the computation for each pair of RNAs (*O*(*L*^4^) to *O*(*L*^6^)), and for the number of times this computation must be performed (*O*(*N*^2^)). In order to reduce the computational cost for each pair, it is practical to separate the calculations into those for intra-molecular base-pairs and those for inter-molecular base-pairs. In the INTARNA [18] program, which is adopted in our pipeline, *accessibility* based on secondary structural energy of each RNA sequence and the hybridization energy between the two RNA sequences are evaluated as an approximation. Another screening that can reduce the total complexity of the computation is to find the mutually reverse complementary *local* pairs of subsequences from among all the pairs of RNA sequences, followed by computing RNA–RNA interactions between those local pairs. This rough screening on huge number of combinations can itself be computationally expensive, but recent progress in computational methods of sequence analysis enables us to avoid the *O*(*N*^2^) combinatorial explosion of the calculations. To ensure that the above screening works effectively, the pipeline first screens each RNA sequence by its accessibility because a subsequence that forms external base-pairs should not form internal base-pairs.

### Summary of the pipeline

**Fig. 1:**
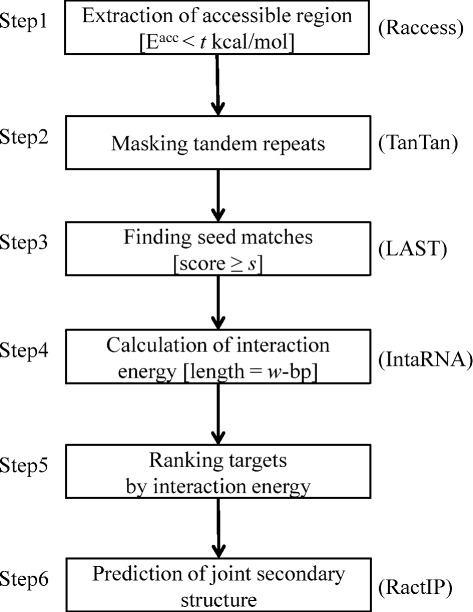
Summary of our pipeline. The programs used for each step are shown in parentheses. Three key parameters (*t*, *s* and *w*) are shown in brackets; these are optimized using verified interaction data for bacteria

A summary of the pipeline is shown in Fig. 1. Given two sets of RNA sequences, where one set is called the *query* RNAs (denoted by *Q*) and the other is called the *target* RNAs (denoted by *T*), our pipeline predicts RNA–RNA interactions between *Q* and *T* (the number of possible candidates of RNA–RNA interactions is |*Q*|×|*T*|). In Step 1, the accessible regions of each RNA sequence are extracted using the RACCESS program [19], and tandem repeats are removed using the TANTAN program [20] in Step 2. For the subsequences screened in Steps 1 and 2, the reverse complementary ‘seed matches’ are detected using LAST [21, 22] in Step 3. In Step 4, the binding energies of pairs of sequences (target and query) around the seed matches are evaluated using INTARNA [18], and candidate interacting pairs are ranked by their binding energy in Step 5. Finally, in Step 6 the joint secondary structures of the interaction site with the minimum interaction energy in each pair of RNA sequences is predicted using RACTIP [23].

### Screening each RNA sequence

First, each RNA sequence is screened to find its inaccessible regions and its tandem repeats, which allows us to extract candidate subsequences to form inter-molecule interactions. This screening requires an *O*(*N*) computation time, where *N* is the number of RNA sequences.

In Step 1, the accessible regions of each RNA sequences are extracted using the RACCESS program [19]; an *accessible* region *r*_*a*_ is a region of length greater than *s* that satisfies *ΔE*^acc^(*r*_*a*_)<*t*, where *s* and *t* are parameters to be specified. Accessibility is defined by

(1) $$\begin{array}{*{20}l} \Delta E^{\text{acc}}(r_{a})=-RT\log(P(r_{a})), \end{array} $$

where *R* and *T* are the gas constant and the temperature, respectively, and

(2) $$\begin{array}{*{20}l} P(r_{a})=\sum_{y\in\mathcal{S}(x)}\exp\left(-E(y,x)/RT\right)/Z(x). \end{array} $$

In Eq. 2, *E*(*y*,*x*) denotes the free energy of secondary structure *y* of an RNA sequence *x*, $\mathcal {S}(x)$ is the set of possible secondary structures of *x* and *Z*(*x*) is the partition function.

For each position in each RNA sequence (in both *Q* and *T*), we compute the accessible energy *E*^*acc*^(*r*_*a*_), where *r*_*a*_ are the *s* consecutive nucleotides starting from the position, using the RACCESS program [19]. (Specifically, RACCESS [19] is executed with the -access_len=*s* option). If *E*^*acc*^(*r*_*a*_)<*t* holds, the positions are considered to be accessible. The parameter *t* in our pipeline is optimized as described in a later section.

In Step 2, tandem repeat regions are masked using the TANTAN program [20] (with the default parameters). This step avoids the explosion of the number of candidates when finding the reverse complementary seed matches in Step 3.

### Seed match

In our pipeline, a seed match means a pair of reverse complementary short subsequences in two RNA sequences (query and target). Specifically, a maximum non-gapped alignment is computed in which the score of G-C and A-U pairs (a.k.a. Watson-Crick base-pairs) is +1 and the score of the other pairs is −1. We consider a seed match whose integer score is more than *s* as a candidate RNA–RNA interaction.

In Step 3, the seed matches are detected using LAST version 250 (http://last.cbrc.jp/) [21, 22], with the options -s0 -j1 -e*s*-m1000000 -l*s*, in which -s0 means LAST detects only matches with reverse complementary sequences, -j1 indicates gapless local alignment is conducted, and -e*s* means that LAST reports those alignments whose score is greater than or equal to *s*. The option -m1000000 is the maximum multiplicity for initial seed match, for which we use a large value because we want to obtain as many seed matches as possible to maintain sensitivity. The option -l*s* indicates that the minimum length of initial matches is at least *s*. The threshold *s* is one of the parameters of the pipeline and was adjusted by using a bacterial dataset in our computational evaluations.

Due to the scoring scheme described above, the seed matches whose lengths are more than *s* are found. Note that LAST employs spaced seeds as the default; it was confirmed by a homology search that this realizes a sensitive seed match [21].

### Evaluation of interaction energy and prediction of the joint structure

In Step 4, the interaction energy, denoted by *E*^*int*^, is computed using the INTARNA [18] program, with the default parameters, applied to the sequence including *w* base-pairs upstream and downstream of each seed match. The integer *w* is one of the parameters in our pipeline, and is optimized using bacterial sequences. Although calculating the optimal interaction energy between two RNA sequences entails heavy computational costs, INTARNA computes the minimum interaction energy between two *local* segments taking into account the approximated accessibility of the target sites [18]. For each pair of a query RNA sequence and a target RNA sequence, our pipeline typically predicts multiple *local* RNA–RNA interactions. In Step 5, those interactions are ranked according to *E*^*int*^, calculated in Step 4. We introduce two ranking methods for the predicted interacting pairs with respect to a query as follows.

- MinEnergy: The minimum interaction energy among the interactions contained in each pair of RNA sequences, computed by IntaRNA. This score is adequate for short RNA sequences or for cases in which the strongest local interaction is dominant.
- SumEnergy: The sum of the local interaction energies that are lower than *x* kcal/mol. This score is adequate for long RNA sequences such as lncRNAs and mRNAs and for cases in which several strong interactions exist.

In Step 6, additionally, the *joint* secondary structure of the strongest interaction site in each pair of RNA sequences is calculated using RACTIP [23]. RACTIP estimates precise internal and external base-pairs by sophisticated integer programming using the principle of maximum expected accuracy [24]. This step does not affect the ranking of the predicted interactions, but information about the structural patterns of the local structures is useful for further analysis of the interactions. The predicted joint secondary structures are stored in our database, which is described later in this paper.

### Computational environment

All the analyses of bacterial sequences were performed on the DELL PRECISION T7500, including 4 Intel Xeon CPU E5620 (2.40GHz, 4-cores). The analyses of Human transcriptome were performed on two computational environments as follows. Steps 3–6 in our pipeline, which require much more computational time than the other steps, were implemented on the *K* computer (http://www.aics.riken.jp/en/k-computer/about/), including 88,128 SPARC64 VIIIfx CPUs (2.0GHz, 8-cores). The other steps were calculated using the Chimera cluster system at AIST, including 176 Intel Xeon E5550 CPUs (2.53 GHz, 8-cores). In our experiments, a part of those cores is utllized (see the ‘Results and discussion’ section for the details).

### Datasets and evaluation methods

#### Experimentally validated RNA–RNA interactions in *E. coli*

As the known interactions for training the parameters of the pipeline, 44 interacting ncRNA–mRNA pairs were taken from [15]. These pairs comprise 17 sRNAs interacting with 37 mRNAs. As non-interacting mRNA targets of those 17 query ncRNAs competing with the 37 mRNAs, we collected mRNA sequences around the start codons, because the ncRNAs are known to interact with the mRNA sequences around the start codons. About 4200 mRNA sequences of 200 bases, 150 base-pairs upstream and 50 base-pairs downstream from the start codons, were taken from the NCBI web site (http://www.ncbi.nlm.nih.gov/nuccore/NC_000913).

#### Human lncRNA and mRNA sequences

We used 23,898 long ncRNA (lncRNA) and 81,814 mRNA sequences obtained by the Gencode project [25] (http://www.gencodegenes.org/releases/19.html). The average and maximum lengths of the lncRNAs were 955 and 91,677, respectively. While most human protein-coding genes have alternative transcripts, the longest mRNA transcripts were selected for each gene in our analysis. As a result, the number of mRNA sequences was reduced to 20,185, and the average and maximum lengths were 3382 and 109,224, respectively.

#### Evaluation methods

In this study, we use the same evaluation method as [15]. Specifically, for each query RNA, its target RNAs are sorted by rank (using either MINENERGY or SUMENERGY), and the number of true positive predictions (denoted by “nTPs” in the following) are counted for all query RNAs with a given rank.

## Results and discussion

### Training parameters using bacterial RNA–RNA interactions

Our pipeline includes three adjustable parameters (cf. Fig. 1 and Materials and methods): (i) the threshold for the accessibility, denoted by *t*; (ii) the threshold of the score of the seeds, denoted by *s*; and (iii) the length *w* of the flanking sequences around the seed region. Flanking sequences of length *w* upstream and downstream around a seed are used for computing the (local) interaction energy. Those three parameters affect both the prediction accuracy and the computation time. For example, smaller values of *t* and larger values of *s* reduce the computational time because the number of candidates decreases. For optimization of those parameters, the known ncRNA-mRNA interactions in *E. coli* (see Section “Datasets and evaluation methods” for the details) were used. In our pipeline, two kinds of ranking method were introduced for screening the predicted RNA–RNA interactions with respect to each query RNA sequence. The MINENERGY method was used as a ranking method for the procedures described in this section because the query RNA sequences in this dataset were short (the average length is 117.2).

We compared the running time and accuracy of our pipeline with those of a method proposed by [15], which was originally used for predicting sRNA–mRNA interactions in bacteria. Among the several pipelines proposed in Richter et al., we adopted the one based on highly accessible seeds, choosing it because its performance was better than or comparable to the other proposed pipelines (see [15] for the details).

Additional file 1: Figure S1 shows the running time for various values of *w* and *s* (Step 1, reduction of candidates by accessibility was skipped). The computation time decreased as *s* became larger and as *w* became smaller, as expected. For most of the combinations of *s* and *w*, our pipeline was faster than Richter’s pipeline, whose running time for the same dataset was 9.27 h.

Additional file 1: Figure S2 shows a comparison of prediction accuracies between our pipeline and Richter’s pipeline. For our pipeline, only combinations of parameters that produce a result at least five-fold faster than Richter’s pipeline are plotted. Among those combinations of parameters, *s*=8 and *w*=20 achieved the best performance on accuracy, which was almost the same as the performance of Richter’s pipeline.

**Fig. 2:**
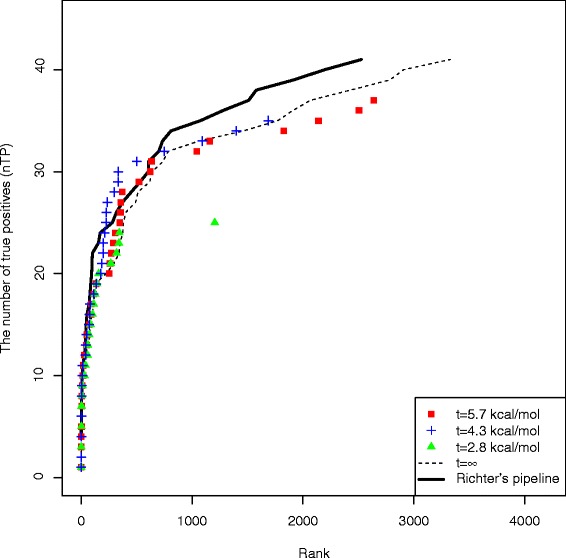
The performance of our pipeline for various values of the parameter *t*, where the parameters *s* and *w* were fixed to be 8 and 20, respectively (cf. Additional file 1: Figure S2). The dashed line for “ *t*=*∞*” shows the performance without considering accessibility

Finally, we evaluated the performance of our pipeline with accessibility filtering by changing the value of *t*, the parameter for the threshold of accessibility, while the other parameters were held fixed at *s*=8 and *w*=20. Figure 2 and Additional file 1: Figure S3 show the performance and the running time, respectively, for various values of *t*. When *t*=4.3 kcal/mol was used, our pipeline (1.00 h) was 9.27 times faster than Richter’s pipeline (9.27 h) while the prediction performances were similar (Figure 2 and Additional file 1: Figure S3). It is interesting that the performance with *t*=4.3 kcal/mol was better than that obtained without using accessibility filtering (Fig. 2).

In summary, when the parameters *s*=8, *w*=20 and *t*=4.3 kcal/mol were used, our pipeline was about 10 times faster than Richter’s pipeline while the performance was almost the same. These optimized values of the parameters, *s*=8, *w*=20 and *t*=4.3 kcal/mol, were used for the comprehensive prediction of RNA–RNA interactions for the human transcriptome, described in later sections.

### Comprehensive predictions for the human transcriptome

We applied our pipeline (cf. Fig. 1) to the human transcriptome and conducted comprehensive predictions of lncRNA–mRNA and lncRNA–lncRNA interactions for the 23,898 lncRNAs (the query) and 44,083 RNAs (the target; including 23,898 lncRNAs and 20,185 mRNAs) in the dataset described in the previous section. The parameters of our pipeline used in these experiments were fixed to *s*=8, *w*=20, *t*=4.3. Overfitting to RNA–RNA interactions in human transcriptome was avoided, because those parameter values were optimized by using an *E. coli* dataset, as described earlier.

The step 1 (accessibility filtering) was performed on the Chimera cluster system using 128 cores, which took 1.16 h. After Step 1, 80.7 % and 79.2 % of the total lengths of lncRNA and mRNA sequences, respectively, were maintained for the next step. The step 2 (tandem repeat masking) was performed on the Chimera cluster system using a single core, which took only about one minute. After Step 2, 78.4 % and 76.7 %, respectively, were maintained. In Step 3, about 4.7×10^10^ seeds were found. In Step 4, we computed the energy for each seed with both upstream and downstream flanking sequences (*w*=20 base-pairs). For steps 3 to 5, 40,000 cores on the K computer (http://www.aics.riken.jp/en/k-computer/about/), one of the fastest computers in the world, were used for the calculation, which took 45 h. For a machine with a single core, however, these processes are estimated to take 1.8×10^6^ h (205 years). This indicates that it is presently infeasible to make comprehensive predictions about RNA–RNA interactions in the human transcriptome without using a supercomputer. (It is noted that Step 3–5 include several pre- and post-processes, whose running time can be neglected compared to the total computational time). Finally, in step 6, the joint secondary structure of the strongest interaction site in each pair of RNA sequences is predicted. For step 6, 16,000 cores on the K computer were used for the calculation, which took 7.3 h.

### Validation of predicted interactions for the human transcriptome

We validated our predicted interactions for the human transcriptome by comparing our predictions with three reliable studies of RNA–RNA interactions [8–10], all of which include experimentally validated lncRNA–mRNA interactions.

#### TINCR–mRNA interactions

Recently, Kretz *et al*. [9] experimentally investigated the interactions between a specific long non-coding RNA, called TINCR, and its target mRNAs using RNA interactome analysis with high throughput sequencing (RIA-Seq). From the 11,225 target mRNAs investigated by Kretz et al. 5195 were included in the mRNA collections of our human transcriptome dataset. Among these, 1062 mRNAs were found to interact with TINCR lncRNA through at least one local RNA–RNA interaction detected as an *enriched segment* by RIA-Seq (an interaction including more enriched segments leads to more reliable TINCR–mRNA interactions; see [9] for the details). Detailed statistics of these mRNAs are shown in (Additional file 1: Table S1).

**Fig. 3:**
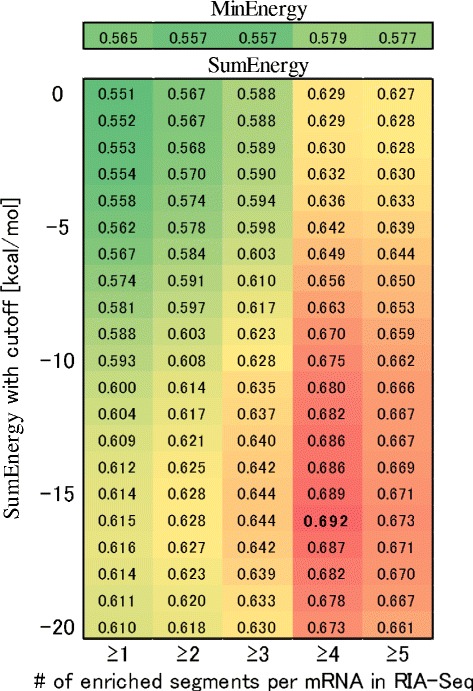
AUC-ROC scores for TINCR–mRNA interactions [9]. The row shows the number of enriched segments per target mRNA in RIA-Seq experiment, which defines the positive dataset; positive datasets with a larger number include more reliable TINCR–mRNA interactions. The column indicates the energy cutoff [kcal/mol] of SUMENERGY in our comprehensive predictions. The bold value is the best score

First, we compared these validated interactions with our predicted interactions, where TINCR (Ensemble ID: ENST00000448587) was included in our human transcriptome dataset. Figure 3 shows an AUC-ROC analysis of our comprehensive predictions of TINCR–mRNA interactions. Among our ranking methods (Step 5), SUMENERGY achieved better performance than MINENERGY. We applied various energy cutoffs to the summation process of SUMENERGY to remove the effect of weak local RNA–RNA interactions with higher energies. The results show that the performance with both a smaller energy threshold and more enriched segments in RIA-seq tends to be better (Fig. 3). These results corresponded to the fact that the validated TINCR–mRNA interactions include several local RNA–RNA interactions. In addition, the best AUC-ROC score of 0.692 (for which the energy cutoff is −16 kcal/mol and the number of enriched windows per mRNA is more than or equal to 4) shows that our prediction pipeline achieved a moderate accuracy by using only sequence information. In the predicted interactions ranked by SUMENERGY with a −16 kcal/mol cutoff (Additional file 1: Table S2), validated TINCR–mRNA interactions consisting of more than three enriched segments were frequently found with better ranking, such as rank1 (ENST00000597346), rank10 (ENST00000301067), rank25 (ENST00000269919), rank37 (ENST00000495893) and rank39 (ENST00000268489).

Finally, we analyzed the pattern of local interactions between TINCR and mRNAs in our predictions. Additional file 1: Figure S4 shows the two predicted patterns of interaction between TINCR and an mRNA: (a) TINCR–mRNA (ENST00000258704), where several local interactions between the segments including TINCR motifs were predicted, and (b) TINCR–mRNA (ENST00000367187), where there are strong interactions between TINCR and the 3 ^′^UTR of the mRNA.

#### 1/2-sbsRNA–mRNA interactions

Recently, Gong and Maquat [8] reported that the lncRNA 1/2-sbsRNA (ENST00000548810) directly interacts with the 3 ^′^UTRs of two mRNAs (SERPINE1 and ANKRD57). These interactions lead to Staufen 1 (STAU1)-mediated messenger RNA decay (SMD) [26].

**Fig. 4:**
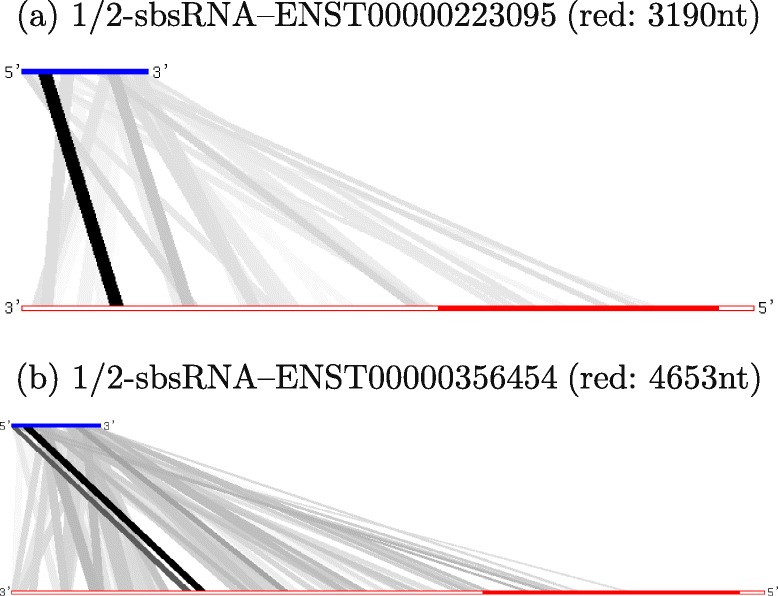
Patterns of predicted local interactions between 1/2-sbsRNA (ENST00000548810) and two mRNAs (ENST00000223095 and ENST00000356454), where darker lines represent more stable interactions whose free energies are smaller. It was reported that both mRNAs interact with 1/2-sbsRNA [8]. The blue and red lines show 1/2-sbsRNA (the left side is 5 ^′^) and mRNA (the left side is 3 ^′^), respectively. In the mRNAs, the 3 ^′^UTR (the left-side) and 5 ^′^UTR (the right-side) are shown as outlined bars

In our predictions with the MINENERGY ranking, the ranks of SERPINE1 (ENST00000223095) and ANKRD57 (ENST00000356454) are 28 (within the top 0.06 %) and 1827 (within the top 4.14 %), respectively, among the predicted interactions with 1/2-sbsRNA (ENST00000548810) as the query. Interestingly, for this lncRNA, ranking with MINENERGY achieved better performance than ranking with SUMENERGY (where the ranks of SERPINE1 and ANKRD57 were 11,296 and 3627, respectively), while the SUMENERGY ranking achieved better performance on TINCR–mRNA interactions. This indicates that ranking by MINENERGY ranking is more appropriate than by SUMENERGY for such interactions. Figure 4 shows the interaction pattern of lncRNA–mRNA, indicating one strong interaction between a region of lncRNA and the 3 ^′^UTR in mRNA. Additionally, the joint secondary structure of the two subsequences (processed by our pipeline) of the two 1/2-sbsRNA–mRNA interactions (predicted by RactIP [23] and shown in Additional file 1: Figure S5) indicates that it includes a long anti-sense-like interaction and the binding sites are located in the 3 ^′^UTR of the mRNAs (Fig. 4). It is also noted that the interaction sites of 1/2-sbsRNA in both interactions are identical.

#### 7SL–TP53 interaction

As described in the introduction, Abdelmohsen *et al.* [10] found that 7SL non-coding RNA interacts with TP53 mRNA, which encodes the tumor suppressor p53. They suggested that 7SL regulates p53 translation by interacting with TP53.

**Fig. 5:**
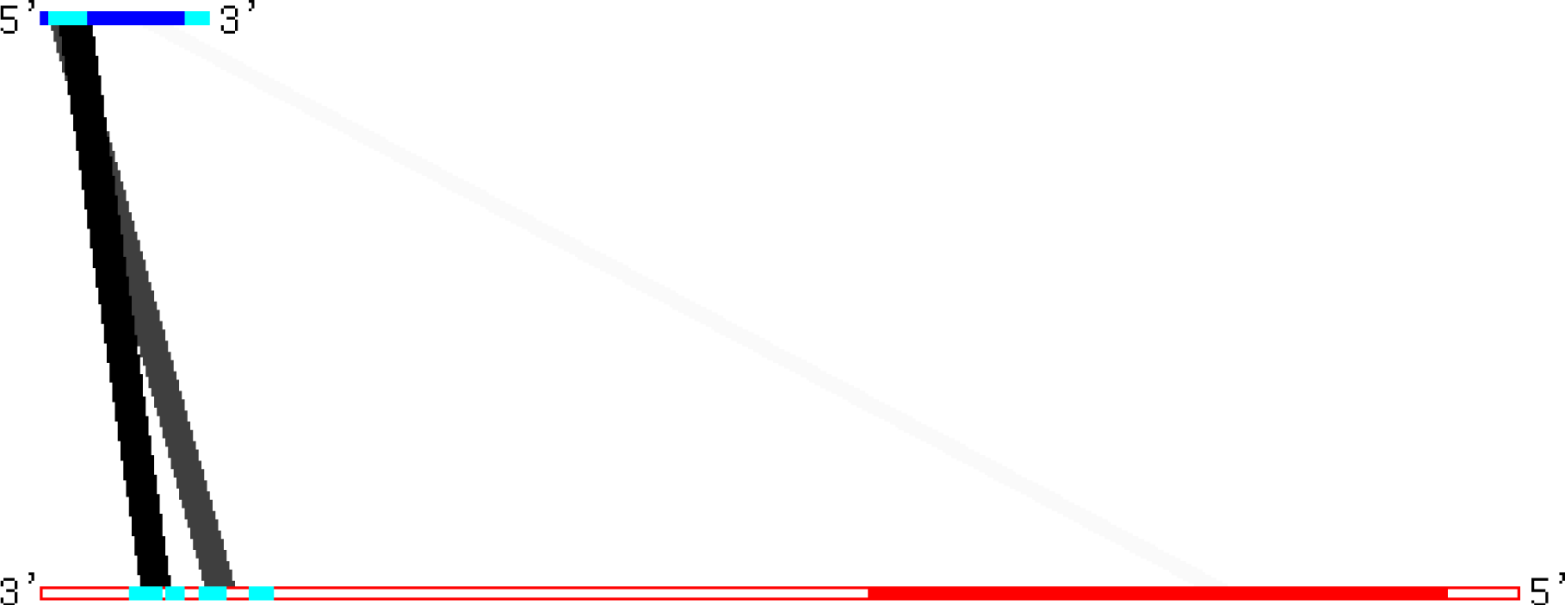
The pattern of the predicted local interactions between 7SL RNA (ENST00000553637) and TP53 mRNA (ENST00000420246). The light blue regions show interaction sites suggested in a previous study [10]

The ranks of 7SL–TP53 interactions in our comprehensive predictions are 2787 for MINENERGY, 2216 for SUMENERGY and 1906 for SUMENERGY with the best energy cutoff. This shows that SUMENERGY is slightly better than MINENERGY. The result is consistent with the results of [10], who suggested multiple interaction sites for 7SL–TP53. In our pipeline, two strong interaction sites between 7SL and the 3 ^′^UTR of TP53 mRNA were predicted (Fig. 5). These two sites are consistent with two of the four interaction sites suggested by Abdelmohsen et al. Interestingly, among the top 100 candidates ranked by SUMENERGY, 71 had the strongest interaction site in the 3 ^′^UTR, suggesting that the 3 ^′^UTR of mRNAs is a common target of 7SL lncRNA.

### An investigation of NEAT1–RNA interactions: a case study of lncRNA–lncRNA interactions

The biological functions of NEAT1 have been well-studied from molecular biology viewpoints (e.g., [7]), with results indicating that NEAT1 is a core molecule forming paraspeckles in the cell. There exist no reports about RNAs (mRNAs or lncRNAs) that directly interact with NEAT1, although several studies of NEAT1–protein interactions have been conducted [27]. In this section, as a case study, we investigate NEAT1–RNA interactions in our comprehensive predictions. Additional file 1: Tables S3 and S4 show lists of NEAT1–RNA interactions predicted by our pipeline, sorted by SUMENERGY and MINENERGY, respectively. (The entire lists are available from our database).

The two lists are dissimilar, indicating that each ranking method captures a different type of RNA–RNA interaction. In the list sorted by MINENERGY (Additional file 1: Table S4), most of the interactions with minimum interaction free energy are located in the 3 ^′^UTR of the mRNAs and the interaction parts in NEAT1 are similar to each other (around position 17,900). In contrast, in the list sorted by SUMENERGY (Additional file 1: Table S3), the rank of the NEAT1–NEAT1 interaction is 20. Although it requires further investigation for validation, there is a possibility that NEAT1–NEAT1 interactions occur in the formation of paraspeckles. Moreover, both lists include many NEAT1–lncRNA interactions, suggesting the possibility that NEAT1 interacts with other lncRNAs.

### Database

**Fig. 6:**
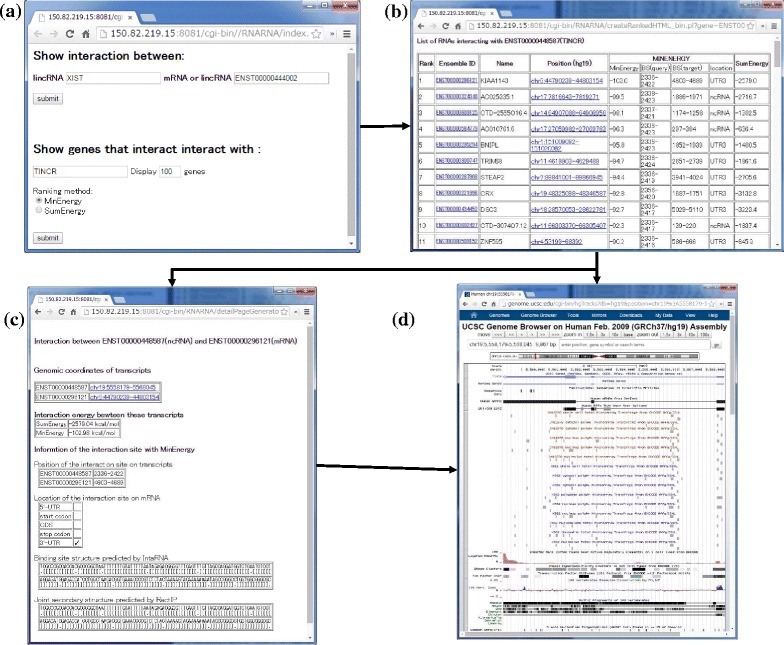
User interface of our RNA–RNA interaction database of lncRNA-mRNA and lncRNA-lncRNA interactions in the human transcriptome. **a** Search window for query by gene name. **b** Search results with MINENERGY and SUMENERGY. **c** Detailed information of predicted interactions, including the joint secondary structure predicted by INTARNA [18] and RACTIP [23]. **d** Genomic context in the UCSC genome browser for target and query RNA sequences, which is opened by clicking on a link in the panel shown in (**b**)

To make our comprehensive predictions of lncRNA–RNA interactions in the human transcriptome publicly available, we have developed a database that contains all the predicted RNA–RNA interactions along with the following information: the rankings by MINENERGY and SUMENERGY, the predicted joint secondary structures, and links to the UCSC genome browser. Figure 6 shows the interface of our database, where users can search by the names (Ensemble ID or HUGO gene symbols) of the lncRNA and mRNA. Each entry includes a link to the UCSC genome browser [28] (http://genome.ucsc.edu/cgi-bin/hgTracks?db=hg19) and users can check the annotation of the target/query RNA sequences in the genomic context. The database is available at http://rtools.cbrc.jp/cgi-bin/RNARNA/index.pl.

### Potential of this study

Our pipeline achieved a performance for bacterial sRNA–mRNA interactions that is comparable to the performance of existing methods (e.g., “Richter’s pipeline”) but with significantly faster calculation. Several promising results were found in the predicted interactions in the human transcriptome in spite of the fact that our pipeline utilizes only sequence information of RNAs. There is room, however, to improve the accuracy of the pipeline and minimize false positive predictions. In the future, we will work to combine our method with additional information, such as expressions (RNA-seq) and localizations. Extensive proteome data for humans [29] and information about protein binding sites in RNA sequences [30] can be also incorporated into those analyses. Moreover, a comparative (consensus interaction-based) approach is promising [16, 31] when homologous sequences are available. Because most lncRNAs are not phylogenetically conserved in distant species, closely related species are needed for the analysis.

In our pipeline, we used the parameters trained using sRNA-mRNAs in E.coli, which might not be optimal for predicting lncRNA-mRNA interactions in human transcriptome. It is, however, interesting that our pipeline achieved modest performance for predicting lncRNA-mRNA interactions with the parameters. This would imply a similarity between the mechanisms of mRNA recognition by sRNA and lncRNA. When the instances of lncRNA-mRNA interactions are accumulated, the similarity and difference between sRNA-mRNA and lncRNA-mRNA interaction will be elucidated. Moreover, it would be interesting to investigate the relation of two ranking methods (MINENERGY and SUMENERGY) with functions of interactions, because using SUMENERGY sometime provided better prediction results than using MINENERGY (TINCR and 7SL lncRNA), whereas the three parameters were optimized for MINENERGY.

Recently, Engreitz et al. have pointed out the importance of interactions between RNA and nascent pre-mRNAs [32]. Although in this study we focused on mature mRNA, it is desirable to make comprehensive predictions of interactions between lncRNA and pre-mRNAs, which requires much greater computational resources than used in this study.

## Conclusion

In this study, we have developed a novel pipeline for predicting RNA–RNA interactions. Our pipeline was tuned and validated using an *E. coli* RNA–RNA interaction dataset and was applied to the human transcriptome using the *K* computer, one of the fastest computers in the world. We compared our predicted RNA–RNA interactions in the human transcriptome with predictions in three existing studies of RNA–RNA interactions. Moreover, we have developed a database to compile predicted interactions, which will be useful for biologists who are interested in specific lncRNAs. To the best of our knowledge, this is the first study to comprehensively predict RNA–RNA interactions in the human transcriptome.

## Additional file

Additional file 1
**Supplementary file (PDF).** This PDF file includes supplmentary texts, figures and tables. (PDF 158 kb)
