# Supplementary material for: Comprehensive prediction of lncRNA–RNA interactions in human transcriptome
Source: BMC Genomics. 2016 Jan 11;17(Suppl 1):12. doi: 10.1186/s12864-015-2307-5 (PMC4895283; doi:10.1186/s12864-015-2307-5)
Supplement: Additional file 1 — Supplementary file (PDF). This PDF file includes supplmentary texts, figures and tables. (PDF 158 kb) [file 12864_2015_2307_MOESM1_ESM.pdf]

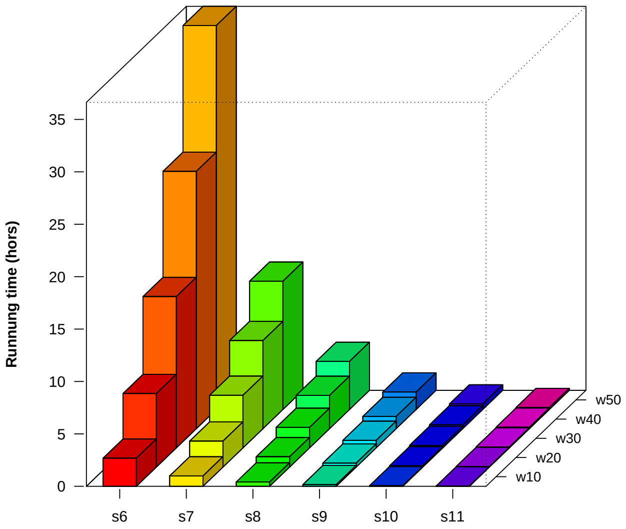

Figure S1 Computation time of our pipeline (Step 2–5) for a bacterial dataset, for various values of the parameters s (the score of seed match) and w (the size of flanking sequence around a seed). In this figure, we show the running time for all combinations of $s = 6, 7, 8, 9, 10, 11$ and $w = 10, 20, 30, 40, 50$. In this experiment, we did not use accessibility to reduce the number of candidates (i.e., the whole region is considered to be accessible). It took 9.27 h when we utilized Richter's pipeline [15] on the same dataset.

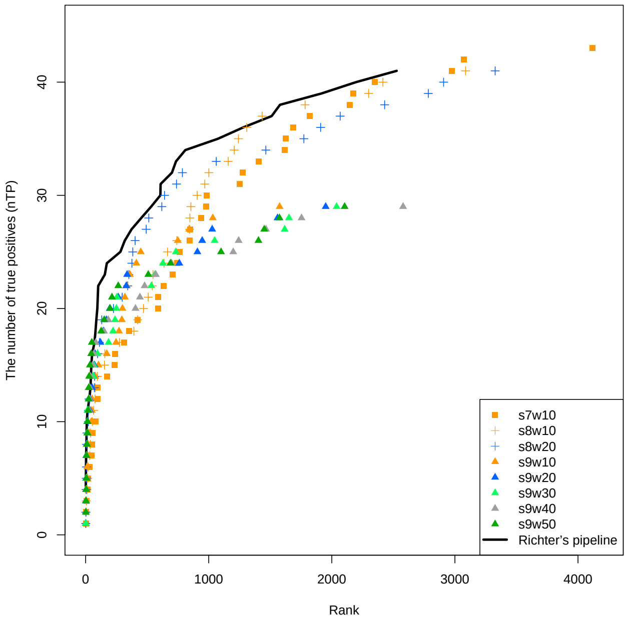

Figure S2 Comparison of our pipeline, for various values of s and w, with Richter's pipeline. Here, the prediction accuracies are shown, limited to parameter sets that result in computation at least five-fold faster than Richter's pipeline when predicting bacterial sRNA–RNA interactions [15]. The dataset includes 44 true positive interactions that are experimentally validated (see the "Materials and Methods" section). In this experiment, we did not use accessibility to reduce the number of candidates (i.e., the whole region is considered to be accessible in this figure). The accuracies for $s = 10$ and $s = 11$ are excluded because of their very low sensitivity.

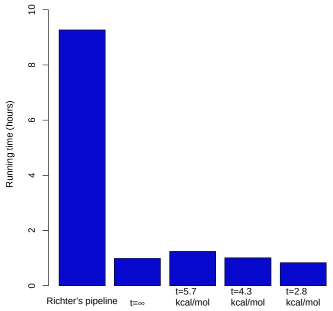

Figure S3 Running time of our pipeline (step 1–5) after reducing the number of candidates by accessibility filtering. The value of t indicates the threshold for accessibility, and $t = \infty$ means no accessibility filtering was performed.

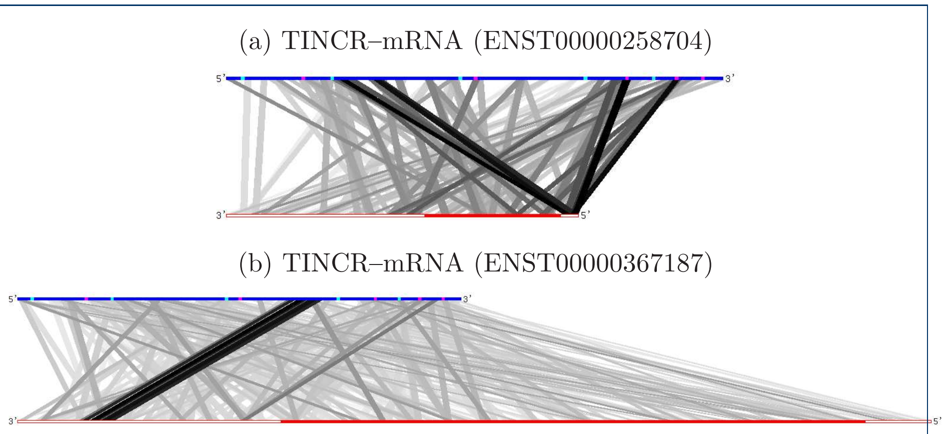

Figure S4 Patterns of predicted local interactions between TINCR (ENST00000448587) and two mRNAs, where darker lines represent more stable interactions whose free energies are smaller. Both interactions were validated by [9]. The blue and red lines show TINCR (the left-side is 5′) and mRNA (the left-side is 3′), respectively. In mRNA, 3′UTR (left) and 5′UTR (right) are shown as outlined bars. In all RNAs, TINCR motifs reported in [9] are shown (anti-sense: light blue; sense: pink). (a) TINCR–mRNA (ENST00000258704), where several local interactions between the segments including TINCR motifs were observed. (b) TINCR–mRNA (ENST00000367187), where strong interactions are predicted in our approach between TINCR and the 3′UTR of the mRNA.

(a) 1/2-sbsRNA (from 75 to 129) and ENST00000223095 (from 2755 to 2809)

```
5' GGAGGATTGCTTGAGCCCAGGAGTTCAAGACCAGCCTGGGCAACATACCAAGACC 3'
   [[[[[[[[[[[[[[[[[[[[[[[[[[[[[[[[[[[[[[[[[[[[[[[.[[[[[[[
3' GGTCTTGCTATGTTGCCTAGGCTGGTCTTGAACTCCTGGGCTCAAGTGATCCTCC 5'
   ]]]]]]].]]]]]]]]]]]]]]]]]]]]]]]]]]]]]]]]]]]]]]]]]]]]]]]
```

(b) 1/2-sbsRNA (from 75 to 129) and ENST00000356454 (from 3458 to 3512)

```
5' GGTGGATCACTTGAGGCCAGGAGTTTGAAACCAGCCTGGCCAACATGGTGAAAAC 3'
   [[.[[[[[[[[[[[[(([[[[[[[[[[[.[[[[[[[[[))[[[[[[[[[[[....
3' GGTCTTGCTATGTTGCCTAGGCTGGTCTTGAACTCCTGGGCTCAAGTGATCCTCC 5'
   ....]]]]]]]]]]]..]]]]]]]]].]]]]]]]]]]]..]]]]]]]]]]]].]]
```

Figure S5 Local joint secondary structures, computed by RactIP [23], between 1/2-sbsRNA and two mRNAs, where a pair of '[' and ']' indicates an external (inter-molecular) base-pair and a pair of '(' and ')' indicates an internal (intra-molecular) base-pair. Both structures are computed for optimal (i.e., minimum free energy) local sequences detected by our pipeline. In each figure, the second sequence is 1/2-sbsRNA and the first sequence is the mRNA.

Table S1 Number of mRNAs interacting with TINCR, included in our dataset, in terms of the number of enriched segments.

# of enriched segments	# of mRNAs
0	4133
1	628
2	243
3	87
4	39
5	26
6	16
7	7
8	2
9	9
10	2
11	1
12	2
Total	5195

Table S2 Ranks of TINCR. The complete list is available from supplementary materials on the journal web site.

rank	Ensembl Transcript ID	sum(energy)	sum(energy) with threshold -16kcal/mol	min(energy)	num_enrich
1	ENST00000597346	-43582.8	-9051.9	-85.0	4
2	ENST00000329627	-10840.9	-5001.1	-79.3	0
3	ENST00000397910	-17865.4	-3559.4	-32.8	0
4	ENST00000501122	-10368.6	-3403.6	-48.7	1
5	ENST00000418539	-7408.0	-3120.4	-80.4	1
6	ENST00000607772	-9266.1	-2854.2	-78.8	0
7	ENST00000422989	-5657.3	-2786.5	-68.1	3
8	ENST00000307602	-7045.1	-2692.3	-75.3	0
9	ENST00000373204	-7009.4	-2581.6	-51.0	1
10	ENST00000301067	-7445.5	-2496.0	-41.5	5
11	ENST00000380358	-4841.6	-2412.4	-40.2	0
12	ENST00000429989	-7192.2	-2374.0	-88.7	1
13	ENST00000309955	-7398.9	-2364.0	-82.0	0
14	ENST00000367255	-14318.4	-2349.5	-37.7	0
15	ENST00000397527	-6914.3	-2340.0	-31.1	0
16	ENST00000415930	-3337.4	-2196.0	-80.0	0
17	ENST00000233190	-5956.1	-2156.9	-68.2	0
18	ENST00000402966	-5011.8	-2139.6	-34.9	0
19	ENST00000361507	-4481.9	-2128.5	-37.4	0
20	ENST00000334801	-4793.3	-2123.7	-44.8	0
21	ENST00000306984	-4233.2	-2120.7	-71.7	0
22	ENST00000171887	-5268.7	-2080.6	-54.9	0
23	ENST00000209875	-5784.9	-2080.2	-74.5	3
24	ENST00000322213	-6338.4	-2069.9	-33.2	0
25	ENST00000269919	-4316.9	-2028.2	-68.9	5
26	ENST00000409535	-5324.0	-2025.0	-53.2	1
27	ENST00000376406	-5426.8	-2020.7	-32.1	0
28	ENST00000369393	-9074.9	-2001.4	-31.6	2
29	ENST00000591344	-4619.0	-1979.0	-75.9	0
30	ENST00000454349	-7457.3	-1974.4	-38.1	0
31	ENST00000409871	-5698.3	-1952.3	-62.9	1
32	ENST00000372037	-5596.3	-1945.3	-77.9	0
33	ENST00000368918	-4679.8	-1886.8	-69.2	0
34	ENST00000339656	-4305.8	-1885.8	-66.9	1
35	ENST00000264065	-8443.1	-1884.8	-62.8	0
36	ENST00000324348	-2716.7	-1884.2	-99.5	0
37	ENST00000495893	-5632.1	-1869.7	-62.4	6
38	ENST00000471389	-6303.3	-1862.6	-72.1	0
39	ENST00000268489	-6644.5	-1849.5	-40.6	6
40	ENST00000299163	-6497.5	-1838.0	-61.3	0
41	ENST00000361127	-5093.3	-1823.8	-70.9	0
42	ENST00000380155	-4175.8	-1792.7	-70.4	0
43	ENST00000406875	-6838.4	-1792.0	-46.2	1
44	ENST00000357137	-3845.7	-1770.2	-69.5	0
45	ENST00000262367	-5003.9	-1761.9	-47.8	0
46	ENST00000377705	-3572.5	-1756.6	-68.9	1
47	ENST00000608999	-7490.0	-1754.9	-64.2	0
48	ENST00000368799	-7847.8	-1751.2	-25.3	0
49	ENST00000337120	-3319.3	-1743.1	-76.1	0
50	ENST00000361354	-8222.4	-1738.8	-69.5	0
51	ENST00000320785	-4283.4	-1737.4	-48.4	3
52	ENST00000261609	-6723.3	-1735.5	-48.3	1
53	ENST00000333891	-9410.0	-1732.6	-33.0	0
54	ENST00000263377	-3470.4	-1728.5	-39.1	0
55	ENST00000378024	-9536.1	-1704.0	-40.2	0
56	ENST00000449592	-3723.0	-1702.7	-66.6	2
57	ENST00000274853	-3128.1	-1697.9	-40.7	0
58	ENST00000262519	-3608.5	-1697.5	-34.8	2
59	ENST00000366577	-5576.7	-1683.5	-70.2	0
60	ENST00000301607	-3584.4	-1674.4	-37.3	6
61	ENST00000262518	-4620.2	-1671.1	-32.4	1
62	ENST00000264033	-5322.1	-1653.7	-76.9	0
63	ENST00000308893	-3084.5	-1644.9	-48.1	0
64	ENST00000300843	-3421.7	-1641.3	-42.0	1

Table S3 List of predicted interaction partners for NEAT1, sorted by SumEnergy.

				MinEnergy				
Rank	Ensemble ID	Name	Position (hg19)	MinEnergy	BS (query)	BS (target)	location	SumEnergy
1	ENST00000597346	KCNQ1OT1	chr11:2629559-2721224	-73.8	17839-17923	67937-68021	ncRNA	-532100.5
2	ENST00000589042	TTN	chr2:179390717-179672150	-47.3	333-382	30983-31032	CDS	-530645.8
3	ENST00000397910	MUC16	chr19:8959521-9092018	-49.9	329-387	24120-24178	CDS	-212080.1
4	ENST00000604411	TSIX	chrX:73012041-73049066	-69.4	17860-17917	649-706	ncRNA	-196345
5	ENST00000397345	NEB	chr2:152341854-152591001	-41	21313-21366	1798-1851	CDS	-141277.8
6	ENST00000370754	DST	chr6:56322788-56819385	-37.3	2186-2236	8661-8711	CDS	-136548.2
7	ENST00000343098	FSIP2	chr2:186603356-186698016	-40.3	4935-4982	196-243	CDS	-135069.1
8	ENST00000609686	GRIN2B	chr12:13693166-14133053	-51.5	8196-8283	7084-7171	UTR3	-133696.1
9	ENST00000367255	SYNE1	chr6:152442820-152958534	-40.1	21316-21374	26923-26981	CDS	-131961.3
10	ENST00000357395	SYNE2	chr14:64319684-64693165	-38	317-367	20837-20887	CDS	-123212.4
11	ENST00000564288	MACF1	chr1:39669919-39952849	-49.4	326-382	466-522	UTR5	-119655.3
12	ENST00000333891	PCLO	chr7:82383330-82792246	-47.5	2169-2237	2495-2563	CDS	-108477.4
13	ENST00000406785	SLC8A1	chr2:40324411-40679209	-47.4	21322-21373	17532-17583	UTR3	-107406.9
14	ENST00000264065	DNAJC10	chr2:183581000-183659191	-67.6	17810-17868	15081-15139	UTR3	-105887
15	ENST00000610020	RPAP2	chr1:92764523-92867613	-70.3	18559-18614	12864-12919	UTR3	-100258.4
16	ENST00000295851	ABI2	chr2:204192943-204312446	-68.2	17784-17867	7963-8046	UTR3	-98940.9
17	ENST00000607772	CNKSR3	chr6:154708639-154831793	-83.9	8254-8456	20188-20390	UTR3	-97887.9
18	ENST00000379442	MUC12	chr7:100612905-100662217	-44.9	2163-2212	15230-15279	CDS	-94520.2
19	ENST00000361354	NCKAP1	chr2:183773844-183903200	-73.7	17816-17872	6623-6679	UTR3	-94156.4
20	ENST00000501122	NEAT1	chr11:65190270-65213011	-54.7	332-389	871-928	ncRNA	-93764.2
21	ENST00000327381	XKR4	chr8:56014950-56454613	-96.5	17869-17950	12315-12396	UTR3	-91741.5
22	ENST00000373191	AGO3	chr1:36396680-36538101	-75.2	17784-17870	4122-4208	UTR3	-90637.1
23	ENST00000367701	ZBTB37	chr1:173838095-173872687	-80.2	19892-19957	8625-8690	UTR3	-90573.8
24	ENST00000359028	AKAP9	chr7:91570190-91739987	-47.5	331-409	139-217	UTR5	-89015.5
25	ENST00000280772	ANK3	chr10:61786057-62149488	-40	341-388	14828-14875	UTR3	-87150.4
26	ENST00000243326	RIF1	chr2:152266456-152338686	-79	17913-17961	10350-10398	UTR3	-86703.1
27	ENST00000360280	VPS13A	chr9:79792362-80036457	-49.3	2170-2224	12415-12469	UTR3	-84804.6
28	ENST00000312675	LPP	chr3:187930722-188608460	-72.3	17784-17870	7832-7918	UTR3	-84767.1
29	ENST00000409195	XIRP2	chr2:167744998-168116263	-37.7	21322-21382	7339-7399	CDS	-83771.8
30	ENST00000609713	KCNJ6	chr21:38979679-39288749	-85.3	17866-17939	16531-16604	UTR3	-83381.3
31	ENST00000526355	GUCY1A2	chr11:106544739-106889250	-40	339-391	7345-7397	UTR3	-82644.5
32	ENST00000355193	KMT2C	chr7:151832015-152133090	-51	330-385	12065-12120	CDS	-82114.5
33	ENST00000382298	SACS	chr13:23902966-24007841	-46	329-380	3800-3851	CDS	-81355.7
34	ENST00000430027	DLX6-AS1	chr7:96584454-96643377	-92.1	17866-17950	232-316	ncRNA	-81303.3
35	ENST00000329627	PEX26	chr22:18560690-18588162	-77.8	19840-19907	10507-10574	UTR3	-81220
36	ENST00000429829	XIST	chrX:73040492-73072588	-47.6	317-387	7215-7285	ncRNA	-81055.6
37	ENST00000261491	DGKH	chr13:42622890-42817032	-59	18620-18683	5422-5485	UTR3	-81030.2
38	ENST00000367941	STX7	chr6:132767007-132834201	-79.4	17839-17923	12946-13030	UTR3	-81017.8
39	ENST00000271588	HMCN1	chr1:185703684-186160081	-35.4	2186-2235	950-999	CDS	-79477.6
40	ENST00000430092	DNAH14	chr1:225117357-225586996	-30.2	2190-2241	13287-13338	CDS	-79364.8
41	ENST00000435803	ABCA13	chr7:48211058-48687091	-69.6	18005-18056	16216-16267	UTR3	-78833.1
42	ENST00000366943	USH2A	chr1:215796237-216596738	-38.1	2156-2217	4465-4526	CDS	-78695.9
43	ENST00000298047	FAT3	chr11:92085263-92629618	-81.2	17842-17917	15233-15308	UTR3	-78467.8
44	ENST00000264501	KIAA1109	chr4:123073489-123283907	-43.1	2164-2220	9469-9525	CDS	-78088.7
45	ENST00000446378	CMYA5	chr5:78985701-79096045	-42.5	2162-2211	2251-2300	CDS	-77632.4
46	ENST00000367607	CEP350	chr1:179923874-180084015	-48.1	333-382	12353-12402	UTR3	-77273.5
47	ENST00000405460	GPR98	chr5:89854618-90460038	-38.5	7389-7436	23-70	UTR5	-77141.3
48	ENST00000463781	MUC4	chr3:195473637-195539148	-54.2	2160-2241	8015-8096	CDS	-76466
49	ENST00000321521	PPIP5K2	chr5:102455905-102548500	-77.4	17784-17867	10895-10978	UTR3	-76251.6
50	ENST00000357033	DMD	chrX:31137346-33229636	-38.9	3225-3273	10831-10879	CDS	-75803.3
51	ENST00000454349	TNRC6B	chr22:40573930-40731811	-62.2	18620-18669	15532-15581	UTR3	-75328.7
52	ENST00000358731	BDP1	chr5:70751443-70863647	-33.3	1690-1745	1721-1776	CDS	-74946
53	ENST00000534358	KMT2A	chr11:118307206-118397539	-50.2	328-380	12644-12696	UTR3	-74047
54	ENST00000389484	LRP1B	chr2:140988993-142889270	-34.8	2148-2195	753-800	UTR5	-73984.3
55	ENST00000393667	GOLGB1	chr3:121382051-121468587	-39.5	323-371	10967-11015	UTR3	-73846.4
56	ENST00000309955	CFLAR	chr2:201980828-202041410	-89.4	17866-17942	11780-11856	UTR3	-73698.7
57	ENST00000310528	SUGT1	chr13:53227002-53275044	-89.5	17784-17872	5824-5912	UTR3	-73680.4
58	ENST00000369393	MDN1	chr6:90352219-90529442	-39.2	2166-2225	7955-8014	CDS	-73400.2
59	ENST00000582970	RNF213	chr17:78234668-78372586	-56.7	2155-2211	18216-18272	UTR3	-72441.9
60	ENST00000327475	DNAH8	chr6:38690623-38998295	-35.7	2174-2229	13108-13163	CDS	-71938.6
61	ENST00000491143	ONECUT2	chr18:55102918-55158529	-51.9	21324-21380	10668-10724	UTR3	-71933.6
62	ENST00000407578	MYCBP2	chr13:77618793-77901177	-36.2	325-386	11198-11259	CDS	-71795.8
63	ENST00000265148	CENPE	chr4:104026964-104119566	-26.7	9044-9106	5122-5184	CDS	-71648.1
64	ENST00000307602	HOOK3	chr8:42752076-42885682	-90.1	17784-17869	5356-5441	UTR3	-71457.9

BS: binding site

Table S4 List of predicted interaction partners for NEAT1, sorted by MinEnergy.

				MinEnergy				
Rank	Ensemble ID	Name	Position (hg19)	MinEnergy	BS (query)	BS (target)	location	SumEnergy
1	ENST00000396872	SLC29A4	chr7:5322562-5346501	-135.4	17839-17950	4777-4888	UTR3	-18954.7
2	ENST00000327535	IFNLR1	chr1:24480648-24513751	-128.4	17855-17944	3905-3994	UTR3	-15120.7
3	ENST00000523336	RP11-317N12.1	chr8:33865376-33896526	-125.6	17842-17949	233-340	ncRNA	-5731.5
4	ENST00000507363	AC008984.2	chr19:54820183-54848439	-116.6	17856-17942	1294-1380	ncRNA	-8113.6
5	ENST00000563540	RP11-146F11.5	chr16:30597229-30619914	-115.8	17839-17947	287-395	ncRNA	-3604.8
6	ENST00000274255	SKP2	chr5:36152170-36184421	-115.7	17856-17949	2823-2916	UTR3	-17096.1
7	ENST00000508072	RP11-425A23.1	chr4:148530704-148538396	-115.6	17839-17948	250-359	ncRNA	-4446.8
8	ENST00000381486	GREB1	chr2:11674243-11782914	-114.3	17860-17950	7297-7387	UTR3	-29141.9
9	ENST00000347108	DZIP1	chr13:96230458-96295517	-114.1	17839-17939	5043-5143	UTR3	-34554.5
10	ENST00000371065	LEPROT	chr1:65886271-65901690	-114.1	17856-17950	4098-4192	UTR3	-20965.7
11	ENST00000322247	FAM210A	chr18:13663347-13726559	-113.9	17860-17949	2523-2612	UTR3	-21105.6
12	ENST00000343450	TADA3	chr3:9824813-9834420	-113.7	17857-17949	2003-2095	UTR3	-9306.5
13	ENST00000309033	UBXN2A	chr2:24163290-24227779	-113.7	17866-17950	3682-3766	UTR3	-27454.7
14	ENST00000337752	C5orf56	chr5:131746674-131798059	-113.6	8256-8428	1573-1745	UTR3	-13145.3
15	ENST00000453655	TRAPPC2	chrX:13730364-13752754	-112.8	17866-17950	1771-1855	UTR3	-13983.7
16	ENST00000366997	LPGAT1	chr1:211916800-212004114	-111.8	17866-17950	2652-2736	UTR3	-36945.3
17	ENST00000412321	RP4-813D12.3	chr20:55841854-55858054	-110.9	17855-17950	501-596	ncRNA	-6864.3
18	ENST00000447393	PACS2	chr14:105781082-105864484	-110.7	17856-17950	4801-4895	UTR3	-16038.6
19	ENST00000592946	AC084219.4	chr19:44609617-44617298	-110.3	9964-10059	2979-3074	ncRNA	-14393.5
20	ENST00000380913	SHROOM2	chrX:9754497-9917483	-109.4	16355-16548	6050-6243	UTR3	-23429.1
21	ENST00000579458	U3	chr18:24267586-24283602	-109.1	17839-17950	1187-1298	ncRNA	-11040.2
22	ENST00000526470	RP11-91I20.3	chr8:61544115-61548830	-109.1	17866-17949	1167-1250	ncRNA	-19346.5
23	ENST00000587791	AC012593.1	chr2:35093462-35399975	-109	17855-17950	409-504	ncRNA	-3112.8
24	ENST00000566293	RP11-438B23.2	chr9:27937616-27944495	-108.8	17855-17947	3162-3254	ncRNA	-35864.5
25	ENST00000305817	PRND	chr20:4702557-4709106	-108.5	17839-17950	3421-3532	UTR3	-17439.4
26	ENST00000369308	LIX1L	chr1:145477086-145501669	-108.3	17860-17939	2940-3019	UTR3	-17976.4
27	ENST00000367996	ADAMTS4	chr1:161154099-161168846	-107.7	17866-17950	3826-3910	UTR3	-37848.3
28	ENST00000547804	LINC00941	chr12:30948866-30955645	-107.7	17873-17949	1352-1428	ncRNA	-9357.6
29	ENST00000392876	NT5DC3	chr12:104166089-104234975	-107.7	17855-17939	4942-5026	UTR3	-27672
30	ENST00000263071	SCARF1	chr17:1537153-1549041	-107.5	17855-17950	3010-3105	UTR3	-9217.4
31	ENST00000354200	TBC1D20	chr20:416125-443197	-107.4	17866-17950	2931-3015	UTR3	-16756.7
32	ENST00000536898	AC002511.1	chr19:35896510-35907742	-106.9	17866-17950	1705-1789	ncRNA	-14987.1
33	ENST00000302692	SLC25A33	chr1:9599542-9645237	-106.5	17862-17947	3193-3278	UTR3	-14859.7
34	ENST00000230882	GHR	chr5:42423880-42721979	-106.2	17865-17950	3723-3808	UTR3	-22028
35	ENST00000336868	NXN	chr17:702554-883010	-106	17866-17947	2334-2415	UTR3	-9186.5
36	ENST00000562077	RP11-529K1.2	chr16:70349544-70380650	-105.7	17866-17950	232-316	ncRNA	-9761.1
37	ENST00000570278	RP11-529K1.2	chr16:70367687-70380650	-105.7	17866-17950	232-316	ncRNA	-5924.5
38	ENST00000315249	RFFL	chr17:33333010-33416338	-105.5	17866-17950	2629-2713	UTR3	-27280.6
39	ENST00000399868	RP11-563J2.2	chr10:6319651-6377944	-105.4	17839-17947	2454-2562	ncRNA	-11857.4
40	ENST00000422989	IRGQ	chr19:44088522-44100287	-105.2	17866-17950	2304-2388	UTR3	-36080.8
41	ENST00000504409	RP11-693J15.5	chr12:92860228-92885867	-105	17866-17946	6443-6523	ncRNA	-52921.9
42	ENST00000367862	POU2F1	chr1:167298282-167396582	-104.6	17866-17950	6972-7056	UTR3	-59668.5
43	ENST00000421685	ZNF674-AS1	chrX:46404929-46407843	-104.2	17842-17933	1278-1369	ncRNA	-8181.5
44	ENST00000560886	RP11-184D12.1	chr15:36231369-36266970	-104.1	17856-17949	199-292	ncRNA	-3597.6
45	ENST00000519807	RPS20	chr8:56979855-56987068	-104	17860-17950	1533-1623	UTR3	-8908.4
46	ENST00000592929	CTB-184G21.3	chr19:19512789-19513412	-103.7	17839-17947	16-124	ncRNA	-1868
47	ENST00000435097	FAM83H-AS1	chr8:144823269-144828506	-103.4	17866-17950	2852-2936	ncRNA	-17703.2
48	ENST00000357503	TOR4A	chr9:140172202-140177093	-103.1	17860-17947	3826-3913	UTR3	-6548.1
49	ENST00000504240	RP11-479O16.1	chr5:58037409-58080606	-102.7	17866-17950	150-234	ncRNA	-2467
50	ENST00000393549	IKBKG	chrX:153775854-153796782	-102.6	17866-17949	2401-2484	UTR3	-18438.6
51	ENST00000374323	SYT15	chr10:46957556-46971400	-102.6	17860-17950	3690-3780	UTR3	-22653.9
52	ENST00000280557	DENR	chr12:123237322-123255611	-102.4	17866-17950	1327-1411	UTR3	-13112.8
53	ENST00000302000	PYGO1	chr15:55831089-55881145	-102.2	17866-17950	4547-4631	UTR3	-42155.4
54	ENST00000356387	ORAI2	chr7:102073997-102097268	-102.1	17866-17947	7040-7121	UTR3	-31056.5
55	ENST00000583062	RP11-258F1.1	chr17:18079880-18088067	-102.1	17866-17950	451-535	ncRNA	-7333.1
56	ENST00000499871	CTD-2201E18.3	chr5:43014837-43018913	-102	17855-17940	64-149	ncRNA	-6823.9
57	ENST00000405954	DUSP28	chr2:241499720-241503431	-101.8	17860-17939	2775-2854	UTR3	-12992.7
58	ENST00000257575	RNFT2	chr12:117176097-117289567	-101.8	17865-17950	2079-2164	UTR3	-15650.3
59	ENST00000258439	TMEM127	chr2:96914255-96931732	-101.5	17839-17935	3375-3471	UTR3	-22336.5
60	ENST00000238831	YIPF4	chr2:32502980-32541663	-101.5	17866-17950	9173-9257	UTR3	-54093.5
61	ENST00000520658	CTB-47B11.3	chr5:156807023-156811958	-101.5	17866-17950	109-193	ncRNA	-2568.6
62	ENST00000508443	CTB-47B11.3	chr5:156802750-156811947	-101.5	17866-17950	98-182	ncRNA	-11445.4
63	ENST00000367187	PIK3C2B	chr1:204391757-204459552	-101.4	17866-17950	6964-7048	UTR3	-26284.6
64	ENST00000325885	ASB4	chr7:95115214-95169544	-101.3	9994-10109	3274-3389	UTR3	-20271.1

BS: binding site
